# Causal agency and the perception of force

**DOI:** 10.3758/s13423-015-0960-y

**Published:** 2015-10-09

**Authors:** Ralf Mayrhofer, Michael R. Waldmann

**Affiliations:** Department of Psychology, University of Göttingen, Gosslerstr. 14, 37073 Göttingen, Germany

**Keywords:** Causal asymmetry, Force asymmetry, Causal agency, Michotte task, Physical causality

## Abstract

In the Michotte task, a ball (X) moves toward a resting ball (Y). In the moment of contact, X stops und Y starts moving. Previous studies have shown that subjects tend to view X as the causal agent (“X launches Y”) rather than Y (“Y stops X”). Moreover, X tends to be attributed more force than Y (force asymmetry), which contradicts the laws of Newtonian mechanics. Recent theories of force asymmetry try to explain these findings as the result of an asymmetrical identification with either the (stronger) agent or the (weaker) patient of the causal interaction. We directly tested this assumption by manipulating attributions of causal agency while holding the properties of the causal interaction constant across conditions. In contrast to previous accounts, we found that force judgments stayed invariant across conditions in which assignments of causal agency shifted from X to Y and that even those subjects who chose Y as the causal agent gave invariantly higher force ratings to X. These results suggest that causal agency and the perception of force are conceptually independent of each other. Different possible explanations are discussed.

In Michotte’s (1963) seminal demonstrations of phenomenal causality, subjects observed moving objects that seemingly interacted with one other. For example, in a launching scenario, a ball (X) moves toward a resting ball (Y) and touches it. At this moment, X stops and Y starts moving with the same velocity and direction as X before (see Fig. 1, upper set of pictures, for an illustration). Observers typically describe this scenario as a case in which X launched Y (White, 2006) indicating that X is perceived as the causal agent and Y as the causal patient. Moreover, subjects tend to assume that X exerts more force on Y than vice versa (White, 2009).

**Fig. 1:**
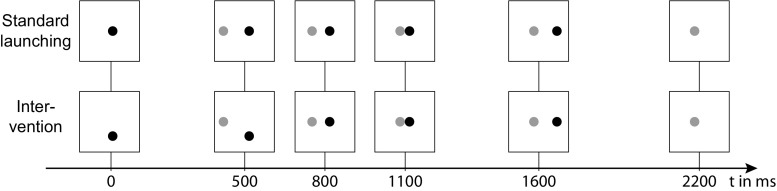
An illustration of the movement patterns of Ball X (gray) and Ball Y (black) depending on movement condition (upper vs. lower set of pictures); see text for details. *Note.* Adapted from “Indicators of Causal Agency in Physical Interactions: The Role of the Prior Context,” by R. Mayrhofer and M. R. Waldmann, 2014, *Cognition,* 132, pp. 485–490

However, this asymmetrical ascription is at odds with physical laws. According to Newtonian mechanics, the physical interaction between two objects X and Y is perfectly symmetric, and the force that X exerts on Y in the moment of collision is equal in magnitude to the counterforce that Y exerts on X. Thus, in purely Newtonian terms, the description that Y stops X is equally consistent with the observation as the description that X launches Y (White, 2006). Moreover, the estimated forces for X and Y should be equal. But what, then, puts us up to such an asymmetric ascription of forces that seemingly violates the underlying symmetry of the physical interaction?

## Causal agency in physical interactions

Although physical interactions themselves are symmetric, a general distinction between a more active object involved in the cause event (i.e., X’s collision with Y) and a more passive object involved in the relevant effect event (i.e., Y being launched) is shared by all theories that address the perception of causal interactions. We are going to use the terms *causal agent* and *causal patient* to distinguish between these different causal roles, but others have suggested a slightly different terminology, for example, *cause object* and *effect object* (White, 2006, 2009, 2012a), *situational agent* and *situational patient* (e.g., Muentener & Carey, 2010), *agentive role* and *receptive role* (e.g., Saxe, Tzelnic, & Carey, 2007), *antagonist* and *agonist* (Talmy, 1988), or *affector* and *patient* (e.g., Wolff, 2007). Causal agents generally refer to objects (including humans) that act on causal patients (see Saxe et al., 2007, for a discussion). Moreover, the assignment of the causal agent and causal patient roles is temporary, restricted to a particular causal interaction; the same object can play the roles of both a causal agent and a causal patient in different interactions. As we will see below, theories of force asymmetry heavily rely on this asymmetrical construal of physical interactions in terms of causal agency.

Typically, the assignment of causal agency in physical interactions is assessed via the linguistic descriptions that subjects choose for describing the scenarios (see, e.g., Mayrhofer & Waldmann, 2014; White, 2006, 2012b). For instance, a frequent description of the Michotte scenario is that “Ball X launched Ball Y,” which assigns X the role of the causal agent (see Dowty, 1991; Mayrhofer & Waldmann, 2015, for discussions of the role of language in assignments of causal agency). If a subject instead chooses the description that “Ball Y stopped Ball X,” this choice would be interpreted as indicating the perception of Y as the more active part in the causal interaction (i.e., that Y acts on X). In our experiments, we have adopted this linguistic measure as an empirical indicator of how subjects assign the roles of causal agents and patients in a particular observed scenario (see also Mayrhofer & Waldmann, 2014).

## Theories of force asymmetry

One of the most prominent theories of causal perception in the Michotte task has been developed by White, who distinguishes between a cause object and an effect object (White, 2006, 2009, 2012a). According to White’s theory, perceived scenes, such as a Michottean launching display, are compared to stored representations of sensomotoric experiences of our actions on objects. During our ontogenetic development, we interact causally with the world by actively and often successfully manipulating objects (e.g., pushing, kicking, squeezing, pulling). We experience our own agency and the force we impose upon the objects. These experiences are stored together with an abstract representation of the actions. When passively perceiving a scene, we compare the movements of the objects with these stored representations. The object whose behavior is most similar to our own actions tends to be viewed as the cause object or causal agent (e.g., the pusher). According to White’s theory, we typically overestimate the force of the cause object relative to the counterforce of the manipulated effect object (i.e., the patient; e.g., the pushed ball) because the (counter-)force exerted by the effect object is perceptually attenuated (White, 2009). Thus, force asymmetry is explained as the result of both (1) an identification of the observer with the causal agent in the interaction via a match to a stored representation of an action that is perceptually similar to the perceived scene and (2) an agent-centered representation of forces in this matched action (i.e., the actor expending more force).

A competing account of how causal interactions are represented has been put forward by Wolff and Shepard (2013; see also Wolff, Ritter, & Holmes, 2014). Whereas White’s (2006, 2009, 2012a) theory is based on the assumption that observers identify themselves with the causal agent, Wolff and Shepard (2013) suggest that it is exactly the other way around: Perceivers “empathize with the object that suffers the effect, that is, the patient” (p. 188). To support their theory, Wolff and Shepard (2013) reported a series of experiments in which subjects observed both causal and noncausal interactions. In the most basic setup, subjects watched a Michottean launching event or, as a noncausal control, a single ball passing from left to right across the screen. Subjects were instructed to immediately press a button after having observed the scenes under the following three conditions: (a) when they heard a sound, (b) when they saw a visual signal, or (c) when they felt a small force applied by means of a haptic controller (as a measure of touch sensitivity). Interestingly, touch sensitivity increased after having observed a causal interaction compared to the noncausal control conditions. By contrast, the type of scene (i.e., causal vs. noncausal) did not influence reaction times to the visual or acoustic stimuli. This dissociation indicates that the perception of causal interactions selectively primes the sensation of touch but does not generally prime motor responses to any kind of stimulus. According to Wolff and Shepard, this is evidence against White’s (2009, 2012a) account: If the match to a stored representation of our own action puts subjects in the role of the causal agent in the scene, one would expect a facilitated motor response to any kind of stimulus when perceiving a causal interaction. Instead, according to Wolff and Shepard, subjects identify with the object playing the role of the patient.

Wolff and Shepard (2013) have not addressed force asymmetry directly in their research. However, since their view represents a contrast to White’s, it is interesting to consider its implications for the issue of force asymmetry. Within the framework of Wolff and Shepard, force asymmetry could be predicted if the assumption is made that the resisting force of the patient with which observers tend to identify is experienced as weaker than the force of the agent. This additional assumption is consistent with Wolff and Shepard’s account, as they say that the patient suffers the effect, which implies that its initial state or tendency has been overpowered by the agent.

## Testing the relation between causal agency and force

Although both accounts attribute the reason for the perception of force in causal interactions differently as either resulting from an identification with the (stronger) causal agent (White, 2012a) or with its (weaker) patient (Wolff & Shepard, 2013), they both imply that force asymmetry arises from the asymmetric assignment of causal roles in a particular causal interaction. Thus, because Ball X is typically assigned the role of the causal agent and Ball Y the role of the causal patient in Michottean launching scenarios, X is attributed more force than Y. Unfortunately, the evidence for a relationship between causal agency and force attribution that has been presented so far is confounded, because factors affecting force attributions have not been independently manipulated from factors that affect intuitions about causal agency. In typical experiments, the physical features of the causal interaction varied across conditions (e.g., the pre- and postcollision velocities; see Hubbard & Ruppel, 2013; White, 2006, 2007, 2009). Since features of the physical interaction at the point of contact of the two balls, such as the pre- and postcontact velocities, may plausibly influence both causal agency and force assignments, the obtained correlation between causal agency and force attributions might be spurious rather than causal.

In order to rigorously test whether assignments of causal agency influence force judgments, it is necessary to manipulate causal agency through features that are independent of the causal interaction itself. We therefore presented variants of the Michotte scenario in which the degree to which the two balls are viewed as causal agents is varied, while the properties of the causal interaction at the point of contact are held constant. According to theories that suggest that force asymmetries are caused by the asymmetric assignment of causal agency, this manipulation should also manifest itself in force attributions. Hence, the more an object is viewed as the causal agent, the stronger its judged relative force should be. To manipulate causal agency independent of the physical interaction, we used a paradigm developed by Mayrhofer and Waldmann (2014; see also Zhou et al., 2012, for a technique of manipulating causal agency through social instead of movement cues).

Mayrhofer and Waldmann (2014) employed variants of the standard launching scenario in which assignments of causal agency shifted from Ball X to Ball Y by manipulating the movement pattern of Y prior to the causal interaction. For instance, in a scenario in which Y was placed below the trajectory of X, then suddenly moved upwards and stopped on X’s path prior to the collision (causing the impression that Y might have intentionally moved into X’s trajectory; *intervention condition*), more people were willing to describe the scene as “Y stopped X” instead of “X launched Y” than in the standard launching scenario.

Because the properties of the causal interaction itself (i.e., the collision) and everything that happened afterwards were held constant across the conditions, these scenarios can be used to test the potential interaction between intuitions about causal agency and force: If the perception of force was a function of causal-agency assignment, force ratings for X and Y should be altered when causal agency is manipulated, even if the collision properties do not vary.

## Experiment 1

In the experiment, we used two variants of the Michottean launching setup, as developed in Mayrhofer and Waldmann (2014). In the standard Michottean launching scenario (see Fig. 1, upper set of pictures) that was also used by White (2006, 2009) and by Wolff and Shepard (2013) Ball X tends to be assigned the role of the causal agent (or cause object) and Ball Y the role of the causal patient (or effect object), as people strongly prefer to describe the scene as “X launched Y” compared to “Y stopped X.” Moreover, X is typically ascribed more force than Y (White, 2009).

In the contrasting *intervention condition*, a sudden movement of Y into X’s trajectory that appears like a self-propelled action shifts the assignment of causal agency from Ball X toward Ball Y. According to theories that claim that force ratings track assignments of causal agency, a similar shift of the force ratings for X and Y should be seen.

### Method

#### Participants and design

Nine hundred thirty-four native speakers of English (58 % female, age range 16–85 years, mean age 48.2 years) were recruited via an online database in the U.K.^1^ and compensated with a voucher worth £0.50. The experiment was run as a 2 (movement pattern: standard Michotte vs. intervention) × 2 (question type: causal-agency vs. force assessment) between-subjects design. Each subject made assessments about both balls (X, Y). Between 233 and 234 subjects were randomly assigned to each condition. We excluded 216 additional subjects (57 % female, age range 17–84 years, mean age 47.6 years) from further analyses because they reported a failure when watching the movie clip or because they failed to pass a simple logical transitivity task that was presented at the end of the experiment (see Procedure section), yielding an overall exclusion rate of 18.8 %.^2^

#### Material

The scenarios were implemented as flash movies that were 720 × 720 pixels in size and played effectively 2,200 ms (preceded and followed by additional 400 ms of black screen). Each movie showed the interaction of two balls, Ball X and Ball Y (one in red and one in blue, each 120 pixels in diameter; see Fig. 1 for an illustration). In Condition 1 (*standard launching*), the standard Michottean setup, Y is at rest in the middle of the screen. Then X enters the scene from the left side on a horizontal trajectory with constant speed until it reaches the center of the screen after 1,100 ms. At the moment of contact, X stops moving and Y starts moving with the same speed as X toward the right hand side of the screen, eventually leaving the scene. In Condition 2 (*intervention*), the position and movement of Y in the first 800 ms was altered: Y was initially at rest in the lower half of the screen (200 pixels above the bottom margin) and started moving upward after 500 ms. Y stopped after 300 ms of movement in the middle of the screen (at exactly the same place as it was placed in Condition 1 at the beginning of the scene) such that Y is at rest 300 ms prior the collision. This manipulation creates an impression of self-propelled movement toward the position in the middle of the screen, and thus the impression of a volitional act aiming to stop X (see Mayrhofer & Waldmann, 2013, 2014, for a detailed discussion). Note that the movement pattern of X was identical in both conditions; the movement pattern for Y was also identical, except for the first 800 ms.

For counterbalancing purposes, we additionally generated an additional movie per movement condition in which we switched the colors of the balls.

#### Apparatus

We had no control over the precise display conditions because the study was run online. However, we ensured that the presented movie fits on the screen under usual display resolutions. At the end of the experiment, 3.74 % of the subjects reported problems when asked whether the movie was played correctly (evenly distributed across conditions); these subjects were excluded from further analyses.

#### Procedure

Subjects were first presented with a brief instruction stating the purpose of the experiment and explaining that they would see a short movie followed by a few questions. After the movie presentation, we asked subjects to make assessments about causal agency or force, respectively (i.e., between subjects). In the *agency-assessment* condition, we requested subjects to rate how well each of two following sentences describes the scene using two separate rating scales ranging from 0 (*not appropriate at all*) to 10 (*highly appropriate*):^3^

1. The red ball launched the blue ball.
2. The blue ball stopped the red ball.

In the *force-assessment* condition, we asked subjects to indicate how much force both balls exerted on each other using two separate rating scales, ranging from 0 (*no force at all*) to 10 (*maximum possible force*):^4^

1. How much force did the blue ball exert on the red ball at the time of collision?
2. How much force did the red ball exert on the blue ball at the time of collision?

Both the two sentences and the rating scales were presented on a single screen; the order of test questions was counterbalanced.

At the end, participants were presented with a simple logical transitivity question—“Imagine four balls: Black, Orange, Yellow, and Green. If Black is bigger than Orange, and Yellow is bigger than Black, and Green is bigger than Yellow, which of the four balls is the smallest?”—along with the four possible answer options presented in randomized order. We used this simple test to exclude subjects who might not have paid sufficient attention to the task.^5^

### Results

#### Control factors

Neither color version of the clip nor the order of the rating scales affected judgments. None of the main effects nor the interaction proved significant (all *p*s > .16). We therefore aggregated over these factors in the following analyses.

#### Assessment of causal agency

**Fig. 2:**
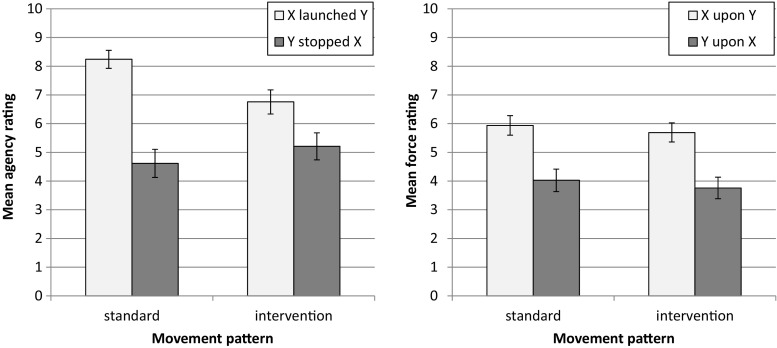
Ratings of causal agency (**a**) and force ratings (**b**) for Ball X and Ball Y per movement condition (standard launching vs. intervention). Error bars indicate 95 % confidence intervals

Figure 2a shows the mean ratings of causal agency for both Ball X and Ball Y, depending on condition (standard launching vs. intervention). The ratings for X were generally higher than the ratings for Y, *F*(1, 465) = 127.4, *p* < .001, η^2^ = .22. As expected, the ratings for X decreased from 8.24 to 6.76 from the standard Michotte condition to the contrasted intervention condition, *t*(465) = 5.5, *p* < .001. The ratings for Y increased accordingly—but to a substantially smaller extent—from 4.61 to 5.21, *t*(465) = 1.7, *p* < .05. This pattern yielded the predicted interaction between condition and rated ball, *F*(1, 465) = 20.6, *p* < .001, η^2^ = .04. The obtained pattern replicates the findings of Mayrhofer and Waldmann (2014) in a between-subjects design and an online paradigm. The results again demonstrate that the manipulation of the movement pattern of Y prior to the causal interaction altered agency attributions.

#### Force assessment

In Fig. 2b, the mean force ratings for Ball X and Ball Y are presented for both conditions (standard launching vs. intervention). The force ratings for X were generally higher than those for Y, *F*(1, 465) = 114.1, *p* < .001, η^2^ = .20. In contrast to the agency ratings, the force ratings did not depend on the movement pattern, *F*(1, 465) = 1.9, *p* = .17. In contrast to the predictions derived from extant theories of force asymmetry, there is no interaction between movement pattern and ball, *F*(1, 465) <1, *p* = .95 (the difference between force ratings is essentially the same for both conditions). This finding shows that force intuitions are not affected by attributions of causal agency (at least if one assumes a linear mapping between causal agency and force ratings).

#### Overall analysis

The separate analyses for causal-agency assignment and the attribution of force revealed that intuitions about causal agency but not force ratings were influenced by the movement patterns of Ball Y prior to the interaction. This dissociation is statistically supported by a three-way interaction between movement pattern, ball, and question type (causal agency vs. force), *F*(1, 930) = 13.0, *p* < .001, η^2^ = .01.

### Discussion

Theories of causal perception suggest that there should be a strong coupling between assignment of causal agency and attributions of force to the involved objects (White, 2012a; Wolff & Shepard, 2013). In contrast to these predictions, we found that force ratings stayed invariant across conditions in which assignments of causal agency, as measured by the standard linguistic measure used in previous research, varied with the movement pattern of Ball Y prior to the collision, but the properties of the interaction itself were held constant. One may object, however, that in both movement-pattern conditions more causal agency was attributed to Ball X, and force asymmetry may only track relative assignments. In such a case, one might not expect a shift in force ratings.

## Experiment 2

In this experiment, we assessed causal agency with a forced-choice measure (i.e., whether either X or Y is the agent; see also Mayrhofer & Waldmann, 2014, Experiment 1) and let participants judge the interacting forces subsequently (i.e., within subject). According to theories of force asymmetry, subjects who chose X as the agent should attribute more force to X and, more importantly, subjects who chose Y as the agent should attribute more force to Y.

### Method

#### Participants and design

Three hundred thirty-six native speakers of English (56 % female, age range 20–87 years, mean age 50.8 years) were recruited as in Experiment 1; 231 additional subjects (64 % female; age range 17 to 83 years; mean 48.5 years) were excluded from further analyses for indicating a movie failure (24 %), for failing to pass the same logical transitivity task as in Experiment 1 (49 %), or for failing to correctly report which Ball eventually left the scene^6^ (55 %), yielding an overall exclusion rate of 40.7 %.^7^ The experiment was run as a 2 (movement pattern: standard Michotte vs. intervention) × 2 (question type: force vs. causal-agency assessment) design. The factor movement pattern was manipulated between subjects, the factor question type within subject. Each subject made agency and force assessments about both balls (X, Y).

#### Material and procedure

We used the same movies and instructions as in Experiment 1.^8^ First, we requested subjects who had watched the movie to choose one of the following sentences as the best description of the scene (i.e., forced choice, counterbalanced order, color-matched; see also Mayrhofer & Waldmann, 2014, Experiment 1):

1. The red ball launched the blue ball.
2. The blue ball stopped the red ball.

Subsequently, we assessed force intuitions as in Experiment 1 but with two separate sliders ranging from 0 to 100 (instead of two separate rating scales) on an additional screen (in counterbalanced order).

### Results

#### Causal agency and force assessments

**Fig. 3:**
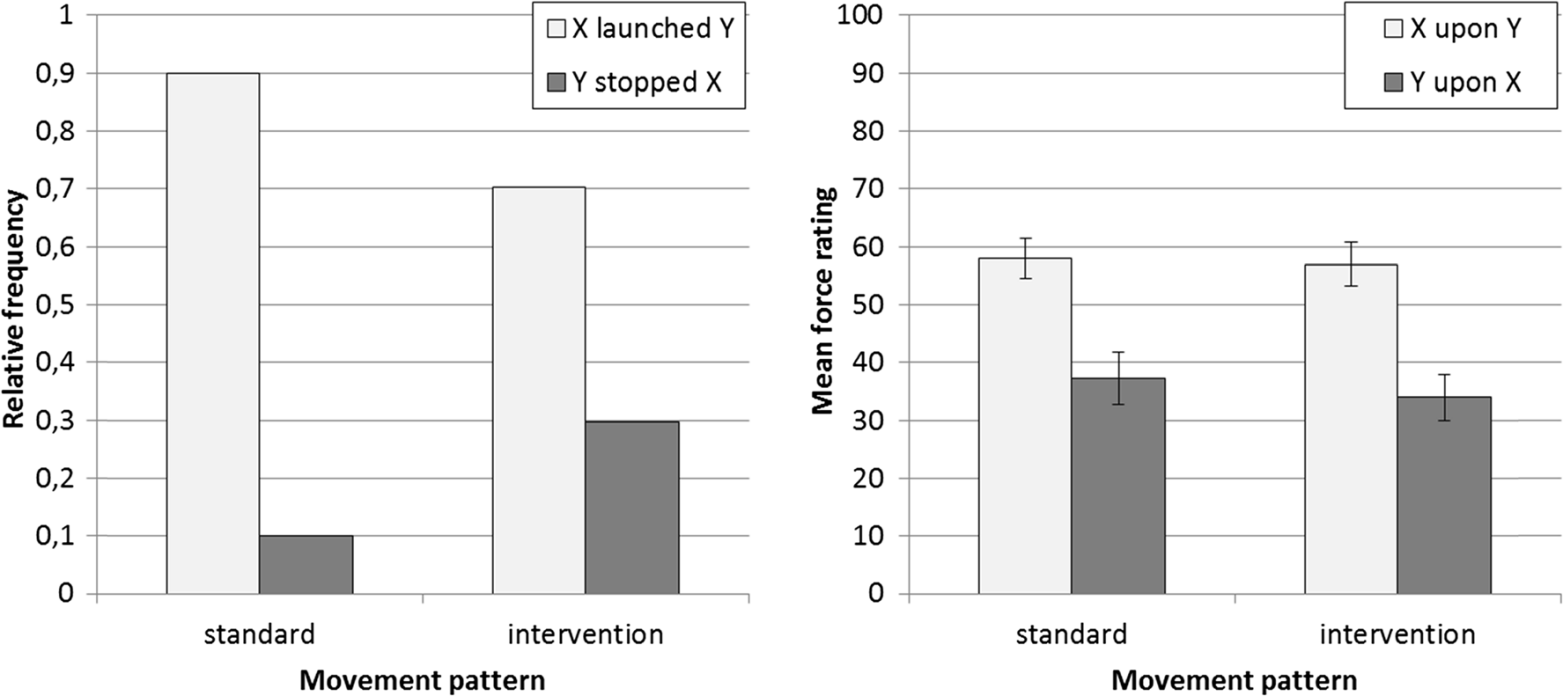
Relative frequency of causal-agency assignments (**a**) and force ratings (**b**) for Ball X and Ball Y per movement condition (standard launching vs. intervention). Error bars indicate 95 % confidence intervals

The willingness to choose Ball Y as the agent increased from 10.1 % in the standard condition to 29.8 % in the intervention condition (see Fig. 3a), ${\chi}_1^2$ = 20.3, *p* < .001.^9^ Hence, the manipulation of the movement properties of Y prior to the causal interaction effectively alters assignments of causal agency.

The force ratings are shown in Fig. 3b. As in Experiment 1, force ratings for Ball X were generally higher than those for Ball Y, *F*(1, 334) = 137.7, *p* < .001, η^2^ = .29. The movement-pattern of Y, however, did not affect force ratings; neither the main effect, *F*(1, 334) < 1, *p* = .33, nor the interaction with the rated ball, *F*(1, 334) < 1, *p* = .54, proved significant.

#### Force assessments conditioned upon assignment of causal agency

As expected, participants who chose Ball X as the causal agent attributed more force to X (57.8) than to Y (33.5), *t*(268) = 12.1, *p* < .001, *d*_*emp*_ = 0.74 (across movement-pattern conditions). However, participants who chose Ball Y as the causal agent also attributed more force to X (56.1) than to Y (43.8), *t*(66) = 2.7, *p* < .01, *d*_*emp*_ = 0.33 (across movement-pattern conditions). This finding is at odds with the assumption that force perception is causally influenced by the assignment of causal agency with more force being attributed to the agentive object of the interaction.

### Discussion

In line with the findings of Experiment 1, we found that a sudden movement of Ball Y into the trajectory of Ball X increased subjects’ willingness to choose Y as the causal agent of the interaction. Force ratings, however, were unaffected by this manipulation—although participants were requested to explicitly assign causal agency prior to the judgments of forces. Additionally, even subjects who chose Y as the causal agent attributed more force to X than to Y which is inconsistent with the hypothesis that asymmetries of force attributions are a consequence of asymmetric agent–patient assignments.

## General discussion

In the standard Michottean launching setup, Ball X is typically viewed as the causal agent or cause object (White, 2006) and is attributed more force than Ball Y (White, 2009). Extant theories of causal perception suggest that the asymmetrical assignment of causal agency to the involved objects is the reason for the asymmetrical attribution of force (White, 2009, 2012a; Wolff & Shepard, 2013). In the present studies, we aimed to directly test this central assumption by manipulating intuitions about causal agency independent of the physical interaction at contact. In contrast to the predictions of the otherwise competing accounts, we found that force attributions were invariant across conditions in which assignment of causal agency was experimentally manipulated. Our findings show that the perception of forces is conceptually independent of intuitions about causal agency.

Our finding leaves us with the open question how force asymmetry could possibly be explained without an appeal to causal agency. Force asymmetry is at odds with theories that model intuitive physics as a case of Newtonian mechanics (e.g., Gerstenberg, Goodman, Lagnado, & Tenenbaum, 2012; Sanborn, Mansinghka, & Griffiths, 2013; Ullman, Stuhlmüller, Goodman, & Tenenbaum, 2014), according to which the forces should be symmetric.

An interesting possibility to reconcile the finding that causal agency is independent of force was raised by a reviewer. Theories connecting force attributions to intuitions about causal agency could be rescued by assuming that there are two causal agents in the scenario: the causal agent prior to the causal interaction that our manipulation influenced and the causal agent at the very moment of contact between objects X and Y. Since we ourselves are sympathetic with dispositional theories (see Mayrhofer & Waldmann, 2015), we agree that this is an interesting possibility. However, this claim is basically derived post hoc from the observed force ratings. Therefore, it remains circular until independent evidence of agency attributions at the point of contact is presented. Also it should be noted that we have used the standard procedure (verbal statements) that had been used previously (e.g., White, 2006, 2012b), and that the statements (e.g., “The blue ball stopped the red ball”) do not only refer to the precollision phase but to the whole event (the blue ball could not possibly be seen as stopping the red ball prior to the collision). We would be eager to see empirical evidence for the claim that agency assignments may shift online during the event. However, in the previous literature this possibility has not been empirically demonstrated.

Causal process theories (e.g., Dowe, 2000; Salmon, 1984) may be an alternative candidate for explaining force asymmetry. Process theories, which seem particularly suitable for modeling physical processes as presented in Michottean scenarios, describe the world in terms of intersecting world lines that embody causal processes. According to Dowe (2000), processes carry a quantity, such as linear momentum, mass-energy, or charge, which is conserved within the process. Of course, only experts know these physical quantities whereas most subjects do not have deep knowledge about physics (see Rozenblit & Keil, 2002). However, despite the lack of elaborate physical domain knowledge it seems plausible to assume that even laypeople represent the Michotte task as a causal process in which some sort of hidden property, a placeholder for the underlying quantity, is transmitted when X moves toward Y and makes contact. A psychologically plausible candidate for such a property might be the (pre-Newtonian) concept of impetus, which is usually represented as an internal force that keeps an object moving and which can be assumed to be transferred from one object to another in a collision event (see, e.g., Kozhevnikov & Hegarty, 2001; McCloskey, 1983). If force intuitions traced the transference of such impetus (i.e., internal force), one would expect an asymmetrical assignment of forces that expresses the directionality of the causal interaction. However, in such a case one would also expect low or zero attribution of force to Y, which is at odds with our finding of relatively high ratings for Y. Actually in line with the prediction of process theories, White (2007, 2009) found near-zero ratings of force attributions to Y with very similar material. We suspect that our higher ratings for Y were caused by a small subset of subjects who activated knowledge from school about Newtonian mechanics and therefore gave equal ratings to X and Y.

In sum, there are several theoretical directions that could be developed to explain agency and force intuitions in the Michotte task. Our experiments provide important new constraints for these theories but further research is needed to determine which of them best captures the phenomena.

## Footnotes

1 http://www.maximiles.co.uk

2 In our experience, the quality of data (e.g., proportion of random guessers or subjects that did not read the instructions carefully) depends on what experiment platform the experiment is hosted. Typically, exclusion rates are lower in experiments run in the M-Turk community (see, e.g., Mason & Suri, 2012), which is not accessible to researchers outside the U.S.

3 The presented sentences were matched to the respective color version.

4 These were the same rating scales as used by White (2007, 2009).

5 An analysis that included the subjects who failed at this test yielded qualitatively the same results (i.e., patterns of statistically significant effects) as those reported below.

6 We added this question because it is central that subjects do not confuse the two balls when selecting the causal agent and rating the forces.

7 Without the additional question about the ball leaving the scene the exclusion rate would have been 37.2 %. We suspect that the reason for the increase (compared to Experiment 1) is the increased length of the experiment such that a higher proportion of subjects lost interest. (Note: Subjects were paid independent of answers, and in contrast to MTurk, there were no negative long-term consequences for random responding. Moreover, MTurk subjects seem to have more experience as subjects of psychological experiments.) As with Experiment 1, we obtained qualitatively the same patterns of results when we included the excluded subjects in our analyses.

8 In Experiment 2, the assignment of colors to the balls was not counterbalanced but randomized across subjects.

9 The preference for selecting Y as the causal agent in the intervention condition is smaller than in the corresponding experiment of Mayrhofer and Waldmann (2014). This might be due to the within-subject design used in the previous study, which may have made the difference between conditions more salient for participants.
